# General practice and the Medical Licensing Assessment

**DOI:** 10.3399/bjgp22X720905

**Published:** 2022-10-30

**Authors:** Douglas GJ McKechnie, Neelam Parmar, Sarah Armstrong, Lucy Pratt, Lindsey Pope, Hugh Alberti, Sophie Park

**Affiliations:** Department of Primary Care and Population Health, University College London, London.; Clinical Lecturer (Teaching) and Deputy Director of Medical Education (Primary Care and Community), Department of Primary Care and Population Health, University College London, London.; GP and iBSc Lead in Primary Care Research and Practice, Department of Primary Care and Population Health, University College London, London.; Patient and public involvement representative.; Director of Community Based Medical Education, School of Medicine, Dentistry & Nursing, University of Glasgow, Glasgow.; Professor of General Practice Education, School of Medical Education, Newcastle University, Newcastle upon Tyne.; Director of Medical Education (Primary Care and Community), Department of Primary Care and Population Health, University College London, London.

From 2024/2025, all UK medical students will sit the Medical Licensing Assessment (MLA),^1^ a mandated national exam comprising: a written applied knowledge test (AKT) in single best answer (SBA) format; and a clinical and professional skills assessment (CPSA). Here we consider the implications for primary care, and for those involved in teaching primary care to medical undergraduates, including GPs and other primary care professionals.

## ASSESSMENT DRIVES LEARNING?

Assessment motivates student learning.^2^ Students often judge the value of learning experiences by their perceived relevance to examinations, and high-stakes assessments such as the MLA are particularly potent influences.^2^ Undergraduate primary care has, historically, suffered in those judgements, with students perceiving that ‘exam-readiness’ is best honed in hospital placements, while primary care placements offer ‘real-world’ medicine — valuable, but not as immediately relevant.^3^ When assessments test, or are perceived to test, certain areas of a medical curriculum and not others, student engagement and learning are potentially unequally incentivised. Furthermore, high-stakes assessments affect how teachers implement their curriculum.^4^ An impetus to ‘teach the test’ is especially likely if institutional rankings are devised based on MLA results, which the General Medical Council (GMC) acknowledges as a possibility,^5^ albeit one they discourage. The MLA may therefore profoundly affect undergraduate medical education, and it is critical that it supports the aims and content of primary care teaching.

## THE MEDICAL LICENSING ASSESSMENT: CONTENT, FORM, AND FUNCTION

Content for both the AKT and CPSA is stipulated in the MLA content map, an evolving document. Assessment materials are to be linked to areas of clinical practice (‘general practice and primary healthcare’), presentations (‘memory loss’), conditions (‘dementia’), clinical and professional capabilities (‘safeguarding vulnerable patients’), and practical skills and procedures. At the time of writing, 163 presentations and conditions were listed under the ‘General practice and primary healthcare’ heading, and many others in the document not under this heading are clearly relevant to primary care (for example, ‘cellulitis’). Much of the MLA’s content, therefore, is already encountered by seeing patients in primary care placements, and primary care is a good setting in which to set clinical scenarios in the MLA (that is, in SBA vignettes or simulated clinical encounters).

While much of the MLA content relates to primary care, the reverse may not be true: much of a primary care clinician’s work is not well represented in the exam. The importance of, and need for, generalist clinician involvement is highlighted in major national healthcare policies such as the NHS Long Term Plan and the Department of Health and Social Care’s 2021 white paper. There are plans afoot to refocus medical education towards the training of expert generalists in Health Education England’s Future Doctor Programme. This is all echoed by the international recognition of the critical role of generalists in health care.^6^ Much work in recent years has articulated the nature of primary care knowledge and generalism, including *Teaching General Practice*,^7^ a national curriculum guide for undergraduate primary care. This eschews a disease-based list of primary care conditions in favour of principles of generalism, such as person- and population-centred care. High-quality generalism embraces, and excels at managing, complexity, multimorbidity, and uncertainty. Skilled generalists still accomplish disease-based management, but this is not necessarily the primary focus of their interactions with patients; rather, to meet patient needs, they are able to integrate, and move between, a range of holistic management approaches. Compartmentalising ‘disease-based’ knowledge in teaching and assessment risks hampering the development of these skills.

Many generalist concepts are difficult to assess within the MLA’s SBA format. SBAs are useful for assessing fact-based recall and application of knowledge, although only to situations and problems in which there is, by definition, a ‘single best answer’. SBAs struggle to test higher-order skills such as analysis, evaluation, and creative problem solving. The CPSA, whose format and delivery are down to individual medical schools, albeit to standards set by the GMC, should assess more complex tasks (usually those involving interaction with a simulated or real patient). However, scoring rubrics for clinical and practical assessments often reward an algorithmic or standardised approach to such tasks,^8^ rather than capturing candidates’ ability to navigate uncertainty, complexity, and probabilistic reasoning. Authentic person-centredness is challenging to test (and promote) within these assessments, with a tendency towards performative, test-wise candidate behaviour to meet tick-box marking.^9^ Superficial (or absent) assessment of person-centredness threatens to reinforce learner perceptions of this as a less valid concept than ‘hard’, objective, ‘fact-based’ knowledge.^10^

The MLA’s stated purpose is to ensure that doctors joining the medical register have *‘met a common threshold for safe practice’*,^11^ thereby reducing variability in assessment standards among UK and international graduates. This could risk hampering institutional autonomy and innovation in teaching and the use of novel assessment methods. Medical schools can still set additional assessments that are required for award of a degree, but it may be challenging to clearly define the role, and relative weighting, of these in relation to the MLA among teachers and students.

## OPPORTUNITIES AND CHALLENGES FOR PRIMARY CARE EDUCATORS

The MLA, therefore, is likely to have substantial effects on undergraduate education: from stimulating curriculum changes to changing how teachers teach and assess, altering student learning behaviour and priorities. There are areas of dissonance between primary care practice and the MLA map. So, how can primary care educators best prepare for its introduction?

We suggest first that primary care educators review the content map and consider how it relates to their existing teaching, and how this is highlighted and communicated to students. Much of the MLA’s content can be taught and assessed within a primary care setting, so we are in a good position to demonstrate the relevance of our specialty to the examination. The MLA content map has three overarching themes: readiness for practice, patient-centredness, and managing uncertainty. Primary care can advance all of these. There are also opportunities for GPs to be involved with the writing, design, and construction of the AKT by joining the various operational groups, for which vacancies are periodically advertised. We suggest that, where possible, all primary care educators consider contributing to local undergraduate assessment as part of their teaching role.

Primary care educators need to consider which elements of the discipline (and their teaching) are not currently assessed within the MLA framework. It is important to be transparent with students about these gaps and discuss the potential value of this knowledge for future practice.

Complementary assessment modalities could be challenging to introduce alongside the MLA, but should be employed to support learning of professionally relevant knowledge (such as generalism). Workplace-based assessments (WPBAs), portfolio-based tasks, quality improvement projects, and reflective essays are already in use. Innovative assessment formats, for example, the Safe and Effective Clinical Outcomes (SECO) exam,^12^ in which students conduct a live simulated consultation, using resources and seeking advice from tutors as required, could better assess students in their ability to provide flexible, situated patient care. Programmatic assessment, using aggregated information from many different multimodal data points collected over time,^13^ may provide a more valid measurement of clinical competence than a one-off examination, and permit regular, meaningful formative feedback for learners.

## CONCLUSION

The MLA is nearing implementation. As a high-stakes exam, it will shape curricula and student/teacher learning priorities. The current MLA presents opportunities to showcase primary care knowledge but potentially undermines some fundamental areas of practice. The involvement of primary care educators is key to maximising the inclusion and visibility of primary care knowledge in the MLA. To enhance opportunities to support student learning about a range of knowledge relevant to professional practice and promote the value of generalism, we must attend to both what is, and is not, included in the MLA, and consider how best to employ and integrate additional assessment formats.
